# Impact of Sidedness of Colon Cancer on Epidemiological, Clinical Presentation, Surgical, Pathological, and Oncologic Outcomes

**DOI:** 10.3390/jpm14121153

**Published:** 2024-12-16

**Authors:** Oswaldo de Moraes Filho, Bruno Augusto Alves Martins, André Araujo de Medeiros Silva, Antonio Carlos Nóbrega dos Santos, Romulo Medeiros de Almeida, João Batista Sousa

**Affiliations:** 1 Coloproctology Service, University Hospital of Brasília, University of Brasilia, Brasília 70840-901, DF, Brazil; bruno.augusto@ebserh.gov.br (B.A.A.M.); andreams2@gmail.com (A.A.d.M.S.); acnobrega@unb.br (A.C.N.d.S.); romuloalmeidadr@gmail.com (R.M.d.A.); sousajb@unb.br (J.B.S.); 2 School of Medicine, University of Brasília, Brasília 70840-901, DF, Brazil

**Keywords:** right colon, left colon, colorectal cancer, clinical presentation, pathological staging, oncological results

## Abstract

**Aim:** The purpose of the study was to identify potential differences between patients with right colon cancer and left colon cancer in epidemiological, clinical presentation, pathological, and surgical results in addition to the impact of the sidedness on disease-free survival (DFS) and overall survival (OS). **Method:** Patients with a diagnosis of colon cancer stages I-IV between 2010 and 2020 were identified from a prospective database in a tertiary single center. Right and left-sided cancer were compared regarding epidemiological, clinical presentation, pathological, and surgical results. Survival analysis was conducted using the Kaplan–Meier method and adjusted hazard ratios for mortality (OS) and disease-free survival (DFS) were obtained using Cox proportional hazards regression. **Results:** The right colon group included 82 (31%) patients and the left colon group 182 (69%). After adjusted analysis, RCC presented less bleeding (RP: 0.31; CI: 0.18–0.56; *p*: 0.0001) and change in bowel habits (RP: 0.60; CI: 0.41–0.87; *p*: 0.0069). A laparotomy approach was more performed in LCC (RP: 0.64; CI: 0.47–0.86; *p*: 0.0029). Regarding pathological results, RCC had more poorly differentiated tumors (RP: 0.81; CI: 0.70–0.94; *p*: 0.05). In the adjusted analysis, there was no difference in survival for right-sided compared to left-sided colon cancer: the hazard ratios were 1.36 (CI 95%: 0.61–3.01; *p*: 0.4490) for OS and 2.04 (CI: 0.91–4.59; *p*: 0.0814) for DFS. **Conclusions:** In this population-based cohort, we found no impact of colon cancer sidedness on OS and DFS. RCC presented less differentiated tumors and LCC presented more bleeding and change in bowel habits.

## 1. Introduction

Colorectal cancer (CRC) is a commonly diagnosed cancer globally, ranking fourth in prevalence and second in global mortality according to the GLOBOCAN database. In 2020, CRC accounted for 1.9 million new cases and 935,000 deaths worldwide [1]. In Brazil, it stands as the second most common cancer for both men and women.

Recent years have seen a growing interest in understanding the distribution of colorectal cancer within the colon’s different segments. The right colon, from the cecum to two-thirds of the transverse colon, originates from the midgut. Conversely, the left colon, from the last third of the transverse colon to the rectosigmoid transition, arises from the hindgut [2].

It seems that this is not just an anatomical difference. In the 1980s and 1990s, Beart et al. and Bufill et al. pioneered the exploration of epidemiological, histopathological, biological, and molecular differences between right and left colon cancer [3,4]. Those findings raised the question of whether right colon cancer must be a different entity from left colon cancer. Furthermore, a lot of data confirm these differences. Some of them are that precursor lesions on the right side often have a flatter morphology, while those on the left side tend to be more polypoid and histologically, right-sided tumors exhibit a higher prevalence of mucinous types and signet ring cell morphology along with a lower degree of differentiation and larger tumor sizes [5,6,7,8,9]. Factors such as advanced age and multiple comorbidities are commonly associated with right-sided colon tumors [10,11].

It is reasonable to conclude that these differences could influence distinct oncogenic pathways. Right colon tumors are linked to mutations in BRAF pathway genes, the hypermethylation of CpG islands, and microsatellite instability. On the other hand, left colon tumors are more frequently associated with mutations in RAS pathway genes, as well as in APC, SMAD4, and TP53 genes, along with chromosomal instability [12,13,14,15]. In this context, tumor location may also impact the choice of adjuvant therapy. For instance, therapies targeting the epidermal growth factor receptor (anti-EGFR), such as cetuximab, are recommended for patients with left-sided tumors harboring wild-type KRAS, while therapies targeting vascular endothelial growth factor (anti-VEGF), such as bevacizumab, are preferred for patients with metastatic right-sided tumors [16]. Even different compositions and concentrations of the microbiota along the colon have been described [17,18,19].

Despite these distinctions, the impact of tumor location on oncological outcomes remains an area of active investigation, with no clear answer. While some studies report no significant differences in overall survival (OS) and disease-free survival (DFS) based on tumor location [10,20,21], others indicate poorer outcomes for right-sided colon cancers, even in stages I, II, and III [9,11,22,23,24,25]. Consequently, whether these differences are universally critical for guiding treatment and follow-up strategies or if outcomes vary across different populations worldwide remains unclear.

The primary goal of our study is to explore the association between tumor sidedness and oncological outcomes, considering various clinical, surgical, and pathological factors within a Brazilian population diagnosed with colon cancer at all stages. This research is conducted at a single center, comprehensively examining these critical factors.

## 2. Materials and Methods

### 2.1. Study Population and Data Collection

This study is a retrospective cohort conducted at a single center, utilizing prospective data from patients with colon cancer who underwent surgery between January 2010 and December 2020 at the University Hospital of Brasília, Brazil. The research adhered to local ethical guidelines. The study included patients aged 18 years and older diagnosed with adenocarcinoma of the colon. Exclusions encompassed those with extraperitoneal rectal cancer, hereditary colon cancer syndromes (familial adenomatous polyposis and Lynch syndrome) meeting Bethesda and Amsterdam II criteria, synchronous tumors, inflammatory bowel disease, and previous colonic resection.

Right colon cancer (RCC) was characterized by tumors located in the cecum, ascending colon, and the first two-thirds of the transverse colon. In contrast, left colon cancer (LCC) encompassed tumors in the last third of the transverse colon, descending colon, sigmoid colon, and rectosigmoid.

Patients were divided into two groups to assess the impact of tumor laterality on epidemiological data in individuals diagnosed with colon adenocarcinoma. The independent variable was tumor location, classified as the right or left colon. The dependent variables compared between the two groups were sex, age at surgery, BMI, American Society of Anesthesiologists (ASA) classification, smoking status, arterial hypertension, and diabetes mellitus. Age was analyzed as the mean and standard deviation and dichotomized into <65 years and ≥65 years for overall survival analyses. The ASA classification was divided into I + II and III + IV. BMI was reported as the mean and standard deviation and dichotomized into <25 and ≥25 for survival analyses.

The impact of laterality on the clinical presentation was assessed by categorizing patients into two groups. The independent variable was tumor location, classified as the right colon versus left colon. The dependent variables compared between the groups were bleeding, abdominal pain, changes in bowel habits, weight loss, and the duration between symptom onset and diagnosis. The time interval between symptom onset and diagnosis (TISD) was defined as the duration from the initial appearance of any colorectal cancer-related sign or symptom to the establishment of a diagnosis. Results were presented as the mean and standard deviation; however, just for Cox regression analysis evaluating disease-free survival and overall survival, TISD was dichotomized into <6 months and >6 months.

Surgical data encompassed the surgical approach, conversion to open surgery, length of hospital stay, operative mortality, reoperation rates, and ICU stay. The conversion variable referred to the unplanned switch from laparoscopy to laparotomy. Operative mortality was defined as death from any cause within 30 days of surgery, with 30 days from the first procedure considered for patients who underwent reoperation. In the multivariate analysis, age (<65 years vs. ≥65 years), BMI, sex, smoking, hypertension, and diabetes mellitus were assessed as potential confounding variables.

Pathological findings included TNM staging, the final stage, total number of lymph nodes harvested, angiolymphatic invasion, perineural invasion, preoperative carcinoembryonic antigen (CEA) levels, and adjuvant chemotherapy status. TNM staging was conducted according to the 9th edition of the American Joint Committee on Cancer (AJCC). The T variable was divided into two groups, namely T1 + T2 and T3 + T4. The N variable was categorized as N0 or N1 + N2 and the M variable. Final staging was grouped into three categories, stages I + II, stage III, and stage IV, and the histological type was classified into two groups, namely well-differentiated + moderately differentiated and poorly differentiated. In the multivariate analysis, age (<65 years vs. ≥65 years), BMI, sex, smoking, hypertension, and diabetes mellitus were considered potential confounders.

Survival outcomes, both overall and disease-free, were compared between patients with right and left colon cancer. The study collected and analyzed data on epidemiological factors, surgical procedures, clinical presentations, pathological characteristics, and oncological outcomes to evaluate the impact of tumor sidedness on these parameters.

### 2.2. Statistical Analyses

For comparison of tumor sidedness occurrence, we used epidemiological, pathological, clinical presentation, and surgical variables. For data presented in frequencies or percentages, we used the chi-square test; for data presented as the mean or standard deviation, we used the t-student or Mann–Whitney tests.

We used Poisson regression models with robust variance (log-linear) and prevalence ratios (PR) with an interval of 95% to analyze the impact of sidedness, controlling for the effects of the covariates sex, age, BMI, smoking, DM, SAH, and ASA. We used the Poisson regression because it provides a better estimate of the prevalence ratios, which in turn represent a more meaningful effects measure for cross-sectional studies [26]. Except for data on the length of stay, with the time interval between the onset of symptoms and diagnosis and with lymph nodes collected, a linear regression was performed with results expressed through Pearson’s partial correlation coefficient with their respective intervals of 95% confidence.

We used Cox regression models and hazard ratios with a 95% interval to analyze the impact of sidedness on overall and disease-free survival, adjusted for a set of clinical covariates. Initially, simple Cox regression models were fitted for each covariate, and those in which the *p*-value was less than 0.25 were included in the multiple Cox regression analysis. Subsequently, adjustments to these variables were made through a process of removing/including variables. Only those covariates with *p* < 0.05 remained in the final model. Next, the independent variable of interest sidedness was included to verify its impact on the time until the occurrence of death or recurrence after adjusting for possible confounders. Lastly, the hazard ratios (HR) and their respective 95% confidence intervals were calculated.

Overall survival and disease-free survival curves for patients with left and right colon cancer were constructed and compared against each other using the log-rank test.

We performed the analysis using SAS 9.4 version SAS 9.4 TS1M8 (9.4 M8). The significance level was set at 0.05.

## 3. Results

A total of 264 patients with a confirmed histologic diagnosis of colonic adenocarcinoma were evaluated. Among them, 82 (31%) presented right-sided tumors and 182 (69%) left-sided colonic cancers. Mean ages were 62.20 ± 11.87 for right-sided CC and 60.13 ± 11.67 for left-sided CC, with no statistical significance. Also, no differences were identified regarding BMI, ASA status, smoking, diabetes, or hypertension. Table 1 shows the results of the demographic data analysis.

1. Clinical Presentation

For the following results, we set the confidence interval (CI) as 95%, unless stated otherwise. Table 2 shows the unadjusted and adjusted Poisson regression results for binary data and linear regression for data expressed as the mean and standard deviation between sidedness and clinical presentation and surgical outcomes. For the clinical presentation, in the unadjusted analysis, the prevalence of bleeding was significantly lower in patients with right colon cancer (PR = 0.31; CI: 0.18–0.56). Similarly, the prevalence of change in bowel habits was significantly lower in patients with right colon cancer (PR = 0.61; CI: 0.42–0.89). In the analysis adjusted for sex, age, BMI, smoking, and DM confounders, PRs practically did not change and assumed values of 0.31 (CI: 0.18–0.56) and 0.60 (CI: 0.41–0.87), respectively. No statistical significance was found between sidedness with abdominal pain (PR = 1.21; CI: 0.97–1.91–; *p*: 0.0968), weight loss (PR = 1.1; CI: 0.79—1.52; *p*: 0.9937), or the time interval between symptom onset and diagnosis (PR = 0.05; CI: −0.10–−0.20; *p*: 0.5140).

2. Surgical outcomes

As for the surgical outcomes, in the unadjusted analysis, the prevalence of operation via laparotomy was significantly lower in patients with right colon cancer (PR = 0.64; CI: 0.48–0.86). In the analysis adjusted for sex, age, BMI, smoking, DM, HAS, and ASA confounders, the PR did not change (PR: 0.64; CI: 0.47–0.86; *p*: 0.0029). The average length of hospital stay was shorter in the RC group (8.28 ± 7.41 days vs. 9.14 ± 10.15 days, *p* = 0.3731), although this difference was not statistically significant. Similarly, no statistically significant differences were observed in the conversion rates (8.79% in RC vs. 7.32% in LC, *p* = 0.5327), the need for reoperation (13.74% in left-sided cases vs. 7.32% in right-sided cases, *p* = 0.0681), admission to an ICU bed during the postoperative period (30.14% in RC vs. 27.95% in LC, *p* = 0.6438), or postoperative mortality (4.88% in RC vs. 4.40% in LC, *p* = 0.7836).

3. Pathological outcomes
4. In terms of pathological staging outcomes, in the unadjusted analysis, the prevalence of poorly differentiated histological types was significantly lower in patients with left colon cancer (PR = 0.82; CI: 0.71–0.95). In the adjusted analysis by the confounders of sex, age, smoking, and BMI, the PR showed a slight decrease with a value 0.81 (CI: 0.70–0.94). The distribution of T stages (T1 + T2 vs. T3 + T4) was comparable between the right and left colons (26.32% vs. 73.68% in RC and 24.24% vs. 75.76% in LC, *p* = 0.2295). Lymph node staging (N0 vs. N1 + N2) also showed no significant differences between sides (56% vs. 44% in RC and 49.04% vs. 50.96% in LC, *p* = 0.3857). Similarly, M staging (M0 vs. M1) was consistent across groups (84.15% vs. 15.85% in RC and 86.81% vs. 13.19% in LC, *p* = 0.4754). The average number of lymph nodes recovered was comparable (23.32 ± 10.75 in RC vs. 23.11 ± 15.51 in LC, *p* = 0.8639). Rates of perineural invasion (34.78% in RC vs. 32.92% in LC, *p* = 0.8705) and angiolymphatic invasion (42.03% in RC vs. 37.89% in LC, *p* = 0.7401) were also similar between groups. The results are in Table 3 Oncological outcomes.

An unadjusted and adjusted Cox regression was used to determine correlation between sidedness and death outcomes. Initially, demographic variables (age, sex, BMI, ASA, smoking), pathological variables (histological type, angiolymphatic invasion, perineural invasion, final stage, number of lymph nodes, CEA, and adjuvant chemotherapy), the clinical presentation variable (duration of symptoms), and the surgical variable (access route) were used. From the adjustment of the bivariate analysis, only the variables of age, ASA, surgical approach, histological type, and staging presented a *p*-value < 0.25 and were included in the multivariate model. After adjusting the multiple Cox regression model, the variables ASA and staging showed a significant association (*p* < 0.05) with time until death. Later, keeping these variables, the independent variable of interest, sidedness, was introduced in the multivariate model, which did not indicate to be statistically significant (*p* = 0.4990; HR = 1.36; CI: 0.61–3.01) after the adjustment of possible confounders. The results are in Table 4.

When disease-free survival was studied, the same variables were initially used to analyze the overall survival. From the adjustment of the bivariate analysis, only the variables of sex, BMI, TISD, CEA, staging, the number of lymph nodes, and adjuvant chemotherapy had a *p*-value < 0.25 and were included in the multivariate model. From the multiple Cox regression model adjustment, the variables BMI, CEA, and the number of lymph nodes showed a statistically significant association (*p* < 0.05) with time elapsed until recurrence. Later, keeping these variables, the independent variable of interest, sidedness, was introduced in the multivariate model, which did not seem to be statistically significant (*p* = 0.0814; HR = 2.04; 95% CI 0.91–4.59) after the adjustment of possible confounders. Table 5 shows the unadjusted and adjusted Cox regression results between sidedness and disease-free survival.

**Figure 1:**
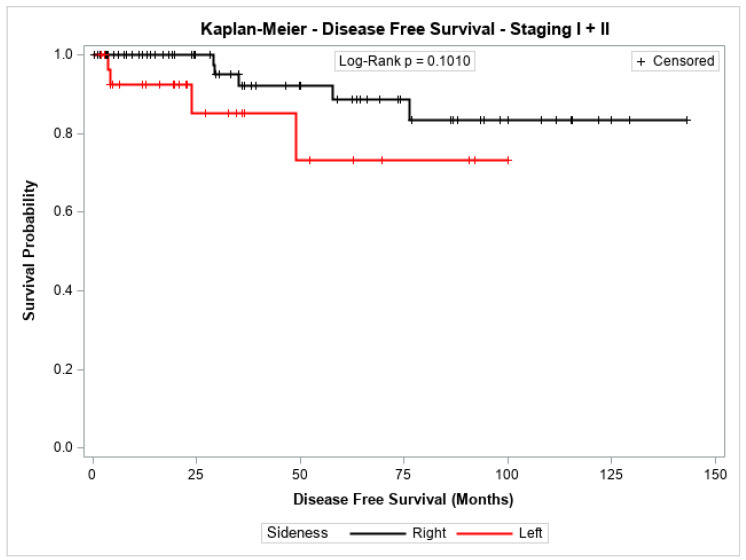
Kaplan-Meier disease-Free survival estimates for patients with right-sided and left-sided colon cancer in stages I + II.

**Figure 2:**
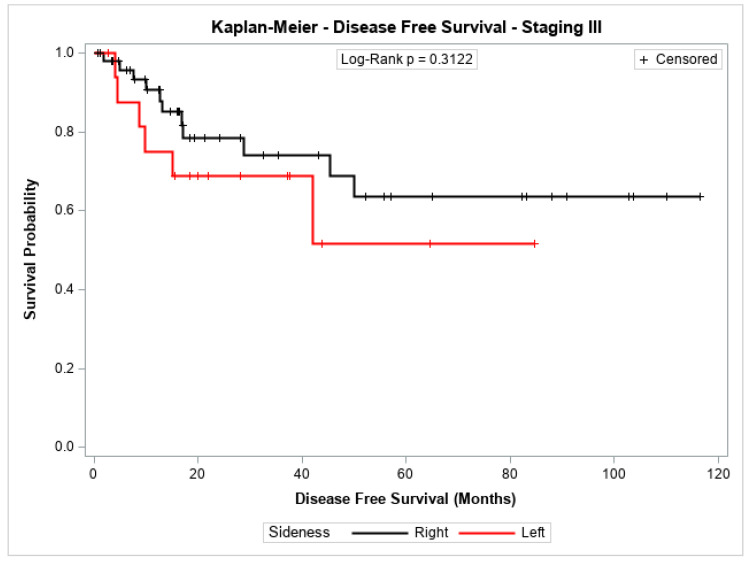
Kaplan-Meier disease-Free survival estimates for patients with right-sided and left-sided colon cancer in stage III.

The probability of disease-free survival for patients with stage I + II or with stage III showed no significant difference between patients with cancer in the left colon compared to those with cancer in the right colon (*p* = 0.1010 and *p* = 0.3122, respectively). Figure 1 and Figure 2 shows the Kaplan–Meier survival curves for disease-free survival at stages I + II and III.

**Figure 3:**
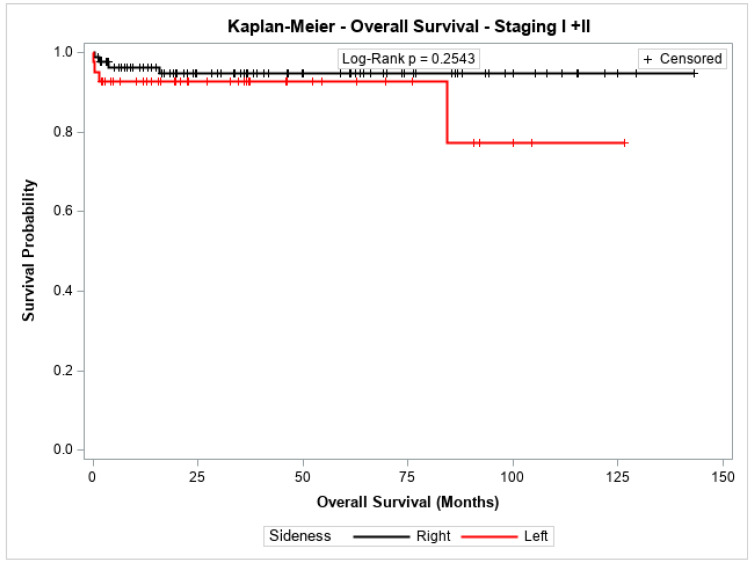
Kaplan-Meier overall survival estimates for patients with right-sided and left-sided colon cancer in stages I + II.

**Figure 4:**
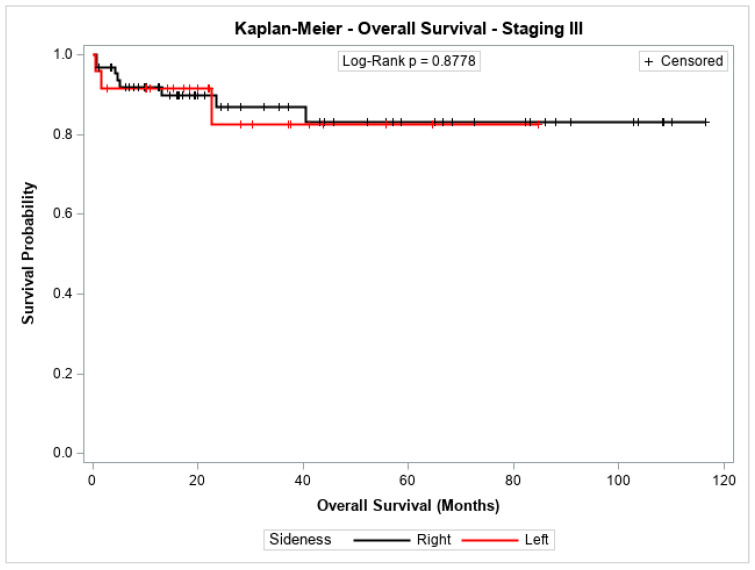
Kaplan-Meier overall survival estimates for patients with right-sided and left-sided colon cancer in stages III.

**Figure 5:**
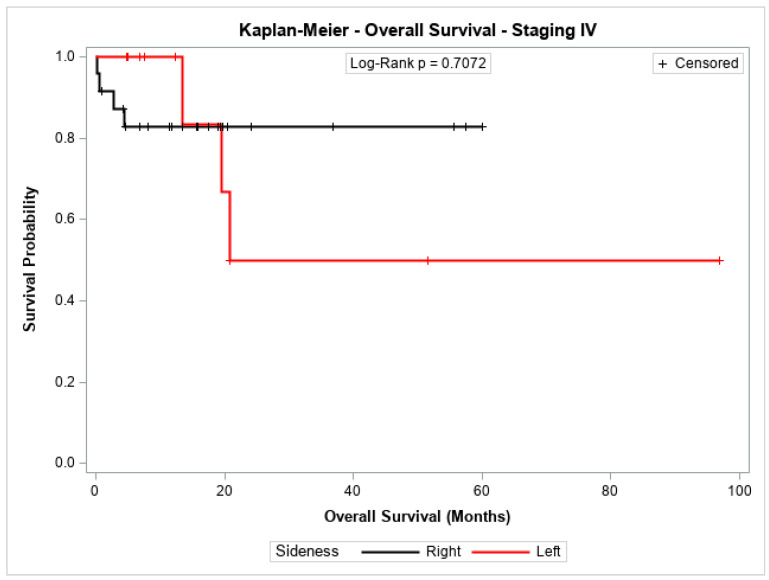
Kaplan-Meier overall survival estimates for patients with right-sided and left-sided colon cancer in stages IV.

The overall survival probability for patients with stage I + II, stage III, or stage IV did not differ significantly between patients with cancer in the left colon compared to those with cancer in the right colon (*p* = 0.2543, *p* = 0.8778, and *p* = 0.7072, respectively). Figure 3, Figure 4 and Figure 5 show the Kaplan–Meier survival curves for overall survival by stages I + II, III, and IV.

## 4. Discussion

In this retrospective cohort study, we analyzed 264 patients diagnosed with adenocarcinoma of the colon and treated at any stage of the disease. The exclusion criteria resulted in a more reliable population of sporadic colorectal cancer patients treated at a surgical colon cancer center, reflecting real-world conditions.

The epidemiological data of this study’s population were quite similar, regardless of the tumor’s location within the colon. However, other researchers have found notable differences between groups based on tumor laterality [27,28,29,30,31]. For instance, Benedix et al. (2010) studied tumor laterality in 17,641 individuals from a German population with colon cancer, and they found that the right-sided group had a significantly higher proportion of older patients, women, and patients with more comorbidities compared to the left-sided group [32]. Additional studies have reported similar findings [6,10,33,34,35].

Similarly, pathological findings were consistent between the two groups, with the exception that right-sided colon cancer (RCC) had a higher proportion of poorly differentiated tumors. However, this did not translate into worse oncological outcomes. Several researchers have studied the relationship between tumor sidedness and pathological features, finding more advanced tumors (pT3 and pT4) and stages on the right side [10,30,36,37,38]. In this cohort, no significant association was observed between sidedness and clinical staging, pT, pN, overall stage, or angiolymphatic or perineural invasions.

Consistent with these findings, survival analysis showed no difference in overall survival between RCC and left-sided colon cancer (LCC) across any group (stages I + II, III, and IV). Additionally, disease-free survival in stages I + II and III was similar. However, the influence of tumor sidedness has been a topic of debate in recent years, with conflicting results reported in the literature.

Since the early studies by Beart et al. and Bufill et al. in the 1980s and 1990s [3,4], which explored sidedness as a potential factor in oncological outcomes and highlighted differences between right and left colon cancers, numerous publications have followed.

Our findings align with several recent studies. In 2017, Karim et al. analyzed 6365 patients with right- and left-sided colon cancer and found no differences in survival outcomes [10]. Similarly, in 2021, Kwak et al. studied 966 non-metastatic patients and reported no significant differences in overall survival or disease-free survival between the two groups [39]. That same year, Malakron et al. also reported similar results, concluding that tumor laterality was not a significant predictor of recurrence in their cohort of 673 patients [40].

In 2024, an integrated analysis of four randomized trials, including 4.113 non-metastatic patients, further reinforced these findings. Dividing the patients into right-sided (N = 1.349) and left-sided (N = 2.764) groups, the study found no significant differences in oncological outcomes [21].

A potential question about these studies is that they focus exclusively on non-metastatic patients. However, Degro et al. (2021) included stage IV patients, as we did, and similarly found no impact of tumor sidedness on overall or disease-free survival across any stage [41].

While these studies support our findings, there is significant evidence suggesting a worse prognosis for right-sided colon cancer compared to left-sided. In a 2015 meta-analysis evaluating 15 studies with a total of 108,474 patients, right-sided tumors were associated with poorer overall survival, though not disease-free survival, even after adjusting for covariates and stratifying by stages I-II [24].

Later, in 2017, Petrelli et al. conducted another meta-analysis, which included 66 studies and 1,437,846 patients. Their analysis found that right-sided colon cancer was a worse prognostic factor for both overall and disease-free survival, even after adjustments and stratification by stage [23]. However, the authors noted limitations in their study, excluding 29 articles where the primary tumor location was not significantly associated with overall survival or hazard ratio data were unobtainable from the publications.

In 2022, Hodges et al. published a study using a propensity score matching method with 26,662 non-metastatic patients in each group (RCC and LCC) and found a worse 5-year overall survival (OS) for right-sided tumors across all stages [42].

The literature shows a high degree of heterogeneity in oncological outcomes. For instance, another meta-analysis found that right-sided colon cancer was associated with improved overall and disease-free survival in stages I and II but a worse prognosis in stage III [42], as Kishiki et al. found in their paper [43].

Although we did not observe significant differences in survival rates between RCC and LCC patients, we did identify certain outcome predictors that impacted mortality differently in each group. The ASA score and tumor staging had a significant effect on overall survival in both groups, highlighting the significant role that comorbidities and cancer stage play in colon cancer mortality.

Similarities were also observed when evaluating surgical outcomes. Despite available data reporting a higher incidence of postoperative ileus and more extended hospital stays following right colectomies [44,45,46], both groups had comparable conversion rates, reoperation, postoperative ICU admission, operative mortality, and length of hospital stay, which aligns with the current literature [38]. In our study, approximately 40% of all surgeries were performed laparoscopically, with a higher proportion in the RCC group (54.8% vs. 36.2%). The reasons for this difference remain unclear, but it could be due to the perception that right-sided laparoscopic colectomies are technically easier than left-sided ones.

Regarding clinical presentation, we found that bleeding and changes in bowel habits were more common in left-sided tumors, consistent with previous findings [6,20,30]. On the other hand, the presence of abdominal pain, weight loss, and the time interval between symptom onset and diagnosis were similar between the two groups. The higher frequency of bleeding in left-sided tumors is likely due to the more distal location of these lesions. At the same time, the narrower lumen of the left colon may account for the increased incidence of bowel habit changes.

Despite these distinctions, oncological outcomes within this specific population were found to be comparable. Establishing a correlation between patients’ geographic location and its impact on these results remains challenging. Nonetheless, this study is significant, given the limited data on this topic from South American and Brazilian populations. Our findings align with those of another Brazilian study, which also reported no significant differences in overall survival between right-sided colon cancer (RCC) and left-sided colon cancer (LCC) [47].

Tumor laterality alone cannot be definitively considered a conclusive factor influencing overall survival and disease-free survival. However, the numerous biological, clinical, and pathological differences between right- and left-sided colon tumors may shape future research directions in colorectal cancer. Future studies should focus on whether these distinctions affect screening, diagnosis, and treatment strategies. For example, investigating the role of the microbiota in oncogenesis and therapy could guide approaches ranging from microbiota modulation for cancer prevention to fecal microbiota transplantation as a therapeutic intervention [48]. Understanding these epidemiological, clinical, and pathological distinctions may pave the way for advancing personalized medicine in managing colorectal cancer.

This study has some limitations. The retrospective character of the study and the reduced sample size are some of them. This may have impacted some results that diverged from the literature. Data referring to alterations in the RAS and BRAF family genes and microsatellite instability research were not carried out in the study due to data unavailability. Perhaps they could demonstrate more differences between the sides. Finally, the mean follow-up time on both sides was not long, being around 3 years. Maybe a more extended follow-up could present different findings.

## 5. Conclusions

In conclusion, the influence of sidedness on colon cancer remains with no clear answer. We found some differences between RCC and LCC in clinical presentation and pathological findings, but there were no differences in overall survival and disease-free survival. The patients with LCC had more bleeding and changes in bowel habits and tumors on the right side of the colon had more poorly differentiated tumors, but the disease laterality had no impact on DFS and OS, even when stratified for stage I–IV.

## Figures and Tables

**Table 1:** Demographic data.

Variables	Total	Right	Left	*p*-Value
**Sex**				
Male	115 (47.6)	30 (36.59)	85 (46.70)	0.1250
Female	149 (52.4)	52 (63.41)	97 (53.30)	
**Age (years)**	60.77 ± 11.75	62.20 ± 11.87	60.13 ± 11.67	0.1866
**BMI (kg/m^2^)**	25.27 ± 4.38	24.59 ± 4.46	25.58 ± 4.33	0.0907
**ASA**				
I + II	199 (70)	57 (69.51)	142 (78.02)	0.1375
III + IV	65 (30)	25 (30.49)	40 (21.98)	
**Smoking**				0.6702
Yes	39 (20.1)	14 (20.59)	25 (18.12)	
No	167 (79.9)	54 (79.41)	113 (81.99)	
**HBP**				0.5578
Yes	120 (53.5)	36 (50.70)	84 (54.90)	
No	104 (46.5)	35 (49.30)	69 (45.10)	
**DM**				0.9782
Yes	48 (23.5)	16 (23.53)	32 (23.36)	
No	157 (76.5)	52 (76.47)	105 (76.64)	

The continuous variables age and BMI were represented as mean and standard deviation and the other variables in frequency (%); BMI = body mass index; ASA = American Society of Anesthesiology Classification; HBP: High Blood Pressure. Data regarding smoking were not available for 58 patients, HBP for 40 patients and DM for 58 patients. The p-value was calculated using the Chi-square or Mann-Whitney test.

**Table 2:** Prevalence Ratio for sideness for clinical presentation and surgical results unadjusted and adjusted for sex, age, BMI smoking, DM, HAS and ASA.

					Non-Adjusted			Adjusted		
**Variables**	Total		Right	Left	PR	CI	*p*-Value	PR	CI	*p*-Value
**TISD**	7.09 ± 6.43		6.85 ± 6.13	7.21 ± 6.58	0.04	−0.11–0.18	0.6279	0.05	–0.10–0.20	0.5140
**Abdominal pain**					1.22	0.97–1.52	0.0847	1.21	0.97–1.52	0.0968
Yes	145 (64.4)		49 (73.13)	96 (60.76)						
No	80 (33.60		18 (26.87)	62 (39.24)						
**Weight loss**					1.06	0.82–1.34	0.6888	1.1	0.79–1.52	0.9937
Yes	122 (52.5)		38 (52.78)	84 (52.50)						
No	110 (47.5)		34 (47.22)	76 (47.50)						
**Bleeding**					0.31	0.18–0.56	<0.0001	0.31	0.18–0.56	<0.0001
Yes	106 (47.6)		13 (19.40)	93 (59.62)						
No	117 (52.4)		54 (80.60)	63 (40.38)						
**Change in Bowel Habit**					0.61	0.42–0.89	0.0106	0.6	0.41–0.87	0.0069
Yes	118 (51.3)		23 (31.94)	95 (60.13)						
No	112 (48.7)		49 (68.06)	63 (39.87)						
**Surgical Approach**					0.64	0.48–0.86	0.0034	0.64	0.47; 0.86	0.0029
Laparotomy	153 ((57.95))		37 (45.12)	116 (63.74)						
Videolaparoscopy	111 (42.05)		45 (54.88)	66 (36.26)						
**Conversion**					0.7	0.23–2.12	0.5327	0.68	0.21–2.16	0.5114
No	242 (91.67)		76 (92.68)	166 (91.21)						
Yes	22 (8.33)		6 (7.32)	16 (8.79)						
**Reoperation**					0.38	0.14–1.07	0.0681	0.41	0.14–1.23	0.1121
No	233 (88.26)		76 (92.68)	157 (86.26)						
Yes	31 (11.74)		6 (7.32)	25 (13.74)						
**Postoperative ICU**					1.12	0.69–1.83	0.6438	0.94	0.59–1.51	0.8027
No	167 (71.37)		51 (69.86)	116 (72.05)						
Yes	67 (28.63)		22 (30.14)	45 (27.95)						
**Perioperative Mortality**					0.83	0.22–3.11	0.7836	0.69	0.19–2.49	0.5697
No	252 (95.45)		78 (95.12)	174 (95.60)						
Yes	12 (4.55)		4 (4.88)	8 (4.40)						
**Length of stay**			8.28 ± 7.41	9.14 ± 10.15	0.06	−0.08–0.20	0.3731	0.09	−0.06–0.23	0.2446

TISD: Time Interval Between Onset of Symptoms and Diagnosis; ICU: Itensive Care Unit. The continuous variable TISD was represented as mean and standard deviation and the other variables in frequency (%). To assess TISD, Pearson’s correlation coefficient was used. TISD: Time interval between onset of symptoms and signs and diagnosis. Data regarding abdominal pain were not available for 39 patients, weight loss for 32 patients, bleeding for 41 patients and change in bowel habits for 34 patients.

**Table 3:** Prevalence Ratio for sideness for pathological Staging not-adjusted and adjusted for sex, age, BMI, smoking, DM, HAS and ASA.

					Non-Adjusted			Adjusted		
**Variables**	Total		Right	Left	PR	CI	*p*-Value	PR	CI	*p*-Value
**Stage T**					0.89	(0.74; 1.07)	0.2295	0.91	(0.76; 1.09)	0.3016
T1 + T2	60	(24.9)	20 (26.32)	40 (24.24)						
T3 + T4	181	(75.1)	56 (73.68)	125 (75.76)						
**Stage N**					0.87	(0.64; 1.19)	0.3857	0.82	(0.59; 1.12)	0.2168
N0	119	(51.29)	42 (56.00)	77 (49.04)						
N1 + N2	113	(48.71)	33 (44.00)	80 (50.96)						
**Clinical Staging**					1.27	(0.66; 2.47)	0.4754	1.31	(0.66; 2.62)	0.4372
M0	227	(85.98)	69 (84.15)	158 (86.81)						
M1	37	(14.02)	13 (15.85)	24 (13.19)						
**Isolated Lymph Nodes**	23.17 ± 14.19		23.32 ± 10.75	23.11 ± 15.51	0.01	(−0.13; 0.15)	0.8639	0.01	(−0.13; 0.16)	0.8573
**Perineural Invasion**					1.03	(0.67; 1.59)	0.8705	1.03	(0.67; 1.61)	0.8767
No	153	(66.52)	45 (65.22)	108 (67.08)						
Yes	77	(33.48)	24 (34.78)	53 (32.92)						
**Angiolymphatic Invasion**					1.06	(0.72; 1.57)	0.7401	1.03	(0.70; 1.52)	0.8884
No	140	(60.87)	40 (57.97)	100 (62.11)						
Yes	90	(39.13)	29 (42.03)	61 (37.89)						
**Staging**					0.91	(0.67; 1.22)	0.5225	0.86	(0.64; 1.17)	0.3441
I + II	125	(50)	41 (53.25)	84 (48.55)						
III	89	(35.6)	24 (31.17)	65 (37.57)						
IV	36	(14.4)	12 (15.58)	24 (13.87)						
**Histological Type**					0.82	(0.71; 0.95)	0.0073	0.81	(0.70; 0.94)	0.0056
Well and Moderately Differentiated	213	(88.75)	57 (77.03)	156 (93.98)						
Poorly Differentiated	27	(11.25)	17 (22.97)	10 (6.02)						
**Adjuvant Chemotherapy**										
No	42	(24.42)	19 (33.93)	23 (19.83)						
Yes	130	(75.58)	37 (66.07)	93 (80.17)						

To assess Isolated Lymph Nodes, Pearson’s correlation coefficient was used.

**Table 4:** Hazard Ratio unadjusted and adjusted for overall survival, by selected demographic and clinical variables.

	Hazard Ratio (IC 95%)			
	Non-Adjusted	*p*-Value	Adjusted ^a^	*p*-Value
	HR (CI)		HR (CI)	
**Age (years)**		0.0514		–
<65	1	-	-	–
≥65	2.05 (1.00; 4.20)	0.0514	-	–
**ASA**		0.0326	-	0.0117
I e II	1	-	1	–
III e IV	2.25 (1.07; 4.74)	0.0326	2.81 (1.26; 6.27)	0.0117
**CEA**		0.0126	-	–
≤5	1	-	-	–
>5	3.44 (1.30; 9.07)	0.0126	-	–
**Surgical Approach**		0.2167	-	–
Videolaparoscopy	1	-	-	–
Open Surgery	1.63 (0.75; 3.55)	0.2167	-	–
**Histological Type**		0.0805	-	–
Well and Moderately Differentiated	1	-	-	–
Poorly Differentiated	2.40 (0.90; 6.42)	0.0805	-	–
**Staging**		0.0251		0.0367
I + II	1	-	1	–
III	2.57 (0.85; 5.25)	0.1088	2.19 (0.88; 5.45)	0.0935
IV	4.13 (1.48; 11.53)	0.0068	3.81 (1.36; 10.71)	0.0111
**Sideness**		0.3261		0.4490
Left	1	-	1	–
Right	1.49 (0.67; 3.28)	0.3261	1.36 (0.61; 3.01)	0.4490

**^a^** Adjusted for ASA, Staging e Sideness. ASA = American Society of Anesthesiology Classification; CEA: Carcinoembryonic Antigen.

**Table 5:** Hazard Ratio unadjusted and adjusted survival free of disease, by selected demographic and clinical variables.

	Hazard Ratio (IC 95 %)			
	Non-Adjusted	*p*-Value	Adjusted ^a^	*p*-Value
	HR (CI)		HR (CI)	
**Sex**		0.1637		–
Male	1	–	-	–
Female	1.66 (0.81; 3.39)	0.1637	-	–
**BMI (kg/m^2^)**	0.88 (0.79; 0.97)	0.0090	0.89 (0.79; 1.00)	0.0440
≥25	1	–	1	–
<25	2.19 (1.07; 4.47)	0.0316	2.19 (1.01; 5.03)	
**TISD**		0.1379	-	–
≤6	1	–	-	–
>6	0.52 (0.22; 1.23)	0.1379	-	–
**CEA**		0.0103		0.0136
≤5	1	–	1	–
>5	2.66 (1.26; 5.64)	0.0103	2.65 (1.22; 5.76)	0.0136
**Staging**		0.0009	-	–
I + II	1	–	-	–
III	3.33 (1.63; 6.79)	0.0009	-	–
**Examined Lymph Nodes**	1.02 (1.00; 1.04)	0.0353	1.03 (1.01; 1.05)	0.0027
**Adjuvance QT**		0.0110	-	–
No	1	–	-	–
Yes	6.42 (1.53; 26.92)	0.0110	-	–
**Sideness**		0.1260		0.0753
Left	1	–	1	–
Right	1.85 (0.84; 4.06)	0.1260	2.08 (0.93; 4.67)	0.0753

**^a^** Adjusted for BMI, CEA, Examied Lymph Nodes and Sideness. BMI: Body Mass Index; TISD: Time Interval Between Onset of Symptoms and Diagnosis; CEA: Carcinoembryonic Antigen.

## Data Availability

The table prospectively filled out at the coloproctology service at the Hospital Universitário de Brasília and used in this article cannot be shared on an online platform due to national data protection laws. The number of the General Personal Data Protection Law (LGPD) is 13,709/2018. The law was sanctioned in 2018 and came into force in September 2020.

## Abbreviations

The following abbreviations are used in this manuscript:

RCC	Right colon cancer
LCC	Left colon cancer
CC	Colon cancer
CRC	Colorectal cancer
OS	Overall survival
DFS	Disease-free survival
IBM	Body mass index
ICU	Intensive care unit
CEA	Carcinoembryonic antigen
ASA	American Society of Anesthesiology
DM	Diabetes Mellitus
